# The influence of the European paediatric regulation on marketing authorisation of orphan drugs for children

**DOI:** 10.1186/s13023-014-0120-x

**Published:** 2014-08-05

**Authors:** Annemarie Rosan Kreeftmeijer-Vegter, Anthonius de Boer, Roselinda H van der Vlugt-Meijer, Peter J de Vries

**Affiliations:** 1 Utrecht Institute for Pharmaceutical Sciences, Division of Pharmacoepidemiology and Clinical Pharmacology, Utrecht University, Utrecht, 3508 TB, The Netherlands; 2 ACE Pharmaceuticals BV, Schepenveld 41, Zeewolde, 3891 ZK, The Netherlands; 3 Department of Internal Medicine, Tergooi Hospital, Van Riebeeckweg 212, Hilversum, 1213 XZ, the Netherlands

**Keywords:** European paediatric drug regulation, Orphan drug development, Paediatric use, Paediatric investigation plan, Rare diseases

## Abstract

**Background:**

Drug development for rare diseases is challenging, especially when these orphan drugs (OD) are intended for children. In 2007 the EU Paediatric Drug Regulation was enacted to improve the development of high quality and ethically researched medicines for children through the establishment of Paediatric Investigation Plans (PIPs). The effect of the EU Paediatric Drug Regulation on the marketing authorisation (MA) of drugs for children with rare diseases was studied.

**Methods:**

Data on all designated orphan drugs, their indication, MA, PIPs and indication group (adult or child) were obtained from the European Medicines Agency (EMA). The outcome and duration of the process from orphan drug designation (ODD) to MA, was compared, per indication, by age group. The effect of the Paediatric Drug Regulation, implemented in 2007, on the application process was assessed with survival analysis.

**Results:**

Eighty-one orphan drugs obtained MA since 2000 and half are authorised for (a subgroup of) children; another 34 are currently undergoing further investigations in children through agreed PIPs. The Paediatric Drug Regulation did not significantly increase the number of ODDs with potential paediatric indications (58% before vs 64% after 2007 of ODDs, p = 0.1) and did not lead to more MAs for ODs with paediatric indications (60% vs 43%, p = 0.22). ODs authorised after 2007 had a longer time to MA than those authorised before 2007 (Hazard ratio (95% CI) 2.80 (1.84-4.28), p < 0.001); potential paediatric use did not influence the time to MA (Hazard ratio (95% CI) 1.14 (0.77-1.70), p = 0.52).

**Conclusions:**

The EU Paediatric Drug Regulation had a minor impact on development and availability of ODs for children, was associated with a longer time to MA, but ensured the further paediatric development of drugs still off-label to children. The impact of the Paediatric Drug Regulation on research quantity and quality in children through PIPs is not yet clear.

## Introduction

Rare diseases are defined as life-threatening or chronically debilitating conditions with such a low prevalence that special combined efforts are needed to ensure adequate medical care. As a guide, a prevalence of less than 5 per 10,000 citizens in the European Union (EU) is considered low [1]. A low prevalence still equals to approximately 250,000 patients in the Community for diseases near the cut-off point. Much rarer diseases only affect a few dozen patients in the whole EU. There are between 5000 and 8000 rare diseases identified so far, affecting an estimated 30 million EU citizens [2]. Over 80% of rare diseases have a genetic background, with the great majority being single-gene defects, although multifactorial and chromosomal defects exist. Other non-genetic rare diseases are due to degenerative and proliferative causes, infectious diseases, treatment-related toxicities, alimentary deficiencies, rare poisonings and injuries [2],[3]. Rare diseases can occur at any age but approximately half of these have their onset at birth or during childhood [4].

Drugs for rare diseases are classified as orphan drugs (ODs). Developing ODs is very challenging. This is mainly due to the various factors that limit clinical studies such as the small number of patients, the heterogeneous and scattered populations, ethical issues (i.e. the use of placebo), lack of validated biomarkers and end-points, poor diagnostics and limited clinical expertise [5], but also by the lack of return of investment in the small target population [6]. To stimulate research, development and placing on the market of ODs, in 2000, incentives were put in place for drug developers, such as a ten-year marketing exclusivity, access to centralised authorisation procedures and fee reductions for regulatory activities (such as protocol assistance, MA applications and inspections) by the European Medicines Agency (EMA) [6]. Another mechanism that may boost the discovery and development of ODs is “repurposing”. This refers to the exploitation of known drugs for new indications [7]. Repurposing receives attention in both the United States and Europe, although differences exist between both continents with respect to policies for repurposing of medicinal products [8]. Several initiatives have been created to identify possible targets for drug repositioning [7]. One of them is the US Food and Drug Administration (FDA) Rare Diseases Repurposing Database to encourage repurposing for rare diseases [9]. There are many examples of ODs that were successfully developed from repurposed drugs [10],[11].

The development of drugs for children with rare diseases poses even more challenges than it does for adults. The biology of the growing child, its changing physiology and psychology are much different from adults and requires research that is dedicated to children [12]. Such research is confronted by technical difficulties and legal and ethical constraints. As a consequence, there is little or no investment in research and development of drugs for the paediatric population. More than half of medicines used for children were never or incompletely studied in this population; their use in children is either unlicensed or off label, i.e. out of the scope of the drug’s authorised label for age, route of administration, dose frequency, formulation or indication [13]. Use of unlicensed drugs or off-label use is especially common for children with rare diseases and is potentially inefficacious and hazardous [14].

The European Regulation (EC) No 1901/ 2006, hereinafter referred to as the ‘Paediatric Drug Regulation’ [1] came into force on 26 January 2007 with the objective to improve the health of European children by facilitating the development, accessibility and safe use of new drugs for children aged 0 to 17 years, through clinical studies. These objectives should be achieved without subjecting children to unnecessary clinical trials and without delaying the authorisation for other age populations. This regulation obliges applicants to submit study results to the EMA for each new medicine, new indication, new route of administration or new formulation, according to an agreed Paediatric Investigation Plan (PIP). This PIP describes the planned paediatric studies and their timelines. It should ideally cover all age groups from birth to adolescence. Paediatric studies may be (partially) ‘waived’ if studies are not feasible, appropriate or safe for (a subset of) the paediatric population or ‘deferred’ if it is appropriate to conduct studies in adults prior to initiating studies in children or if studies in children will last longer than studies in adults. The PIP should also describe the need for the development of age-appropriate formulations and/or additional non-clinical information (such as developmental toxicity studies in juvenile animal). When the PIP is completed and all requirements are met, applicants are rewarded with a six month extension of patent protection. Off-patent products developed exclusively for use in children are granted eight year data- and ten year market exclusivity for the paediatric indication (the Paediatric Use Marketing Authorisation (PUMA)). ODs are rewarded with two additional years of market exclusivity. The Paediatric Drug Regulation was introduced in stages (see Table 1) distinguishing new medicinal products from already authorised medicinal products.

**Table 1:** Implementation phases of the Paediatric Drug Regulation (EC) No 1901/2006

**Category**	**Application**	**Jurisdiction**	**Implementation**
Off-patent medicine	**MA for a paediatric use**	Article 30	26 July 2007
New medicine	**MA that includes a paediatric indication**	Article 7	26 July 2008
On-patent medicine	To include a paediatric indication in an existing MA*	Article 8	26 January 2009

*New indications, pharmaceutical forms and/ or routes of administration which are protected by a supplementary protection certificate or by a patent which qualifies for the granting of a supplementary protection certificate.

All three categories need to comply with the requirements of Article 7: application for MA should include the results of all studies conducted in compliance with an agreed PIP or a decision of the EMA granting a (partial) waiver or deferral [1].

The implementation of the Paediatric Drug Regulation has paid off for non-orphan medicinal products. Five years after its implementation, more medicines have become available for children and more research has been conducted in children [15]. Over 600 PIPs have been agreed upon and 30% of those PIPs include studies with neonates, the most neglected group. In addition, more paediatric clinical trials were conducted and the proportion of clinical trials including children increased over the last 6 years, to approximately 10% [15].

For ODs, the impact of the Paediatric Drug Regulation has not been studied. In this manuscript, we describe the drug application process from orphan drug designation (ODD) to marketing authorisation (MA) and analyse the effect of the Paediatric Drug Regulation on the success rate and time course of obtaining MA.

## Methods

### Orphan drug designations and marketing authorisations

The EMA kindly provided us with a list of all ODDs from 2000 until December 2012 with designation date and number, indication and age category for which the OD is intended (children and/or adults, i.e. potential paediatric use or not). Designations are issued for treatment indications so that drugs with more than one indication occur more than once in this list. Of all ODDs that obtained MA, the European Public Assessment Reports (EPARs) and the product information (Summary of Product Characteristics [SmPC]) available at the EMA website (www.ema.europa.eu) were studied. The following information was extracted: authorisation date, approval conditions (i.e. conditional approval or under exceptional circumstances), indication and age category (children, adults or both) for which the product was authorised. All authorised ODs were cross referenced with the Community register (available at http://ec.europa.eu/health/documents/community-register/html/index_en.htm) of both ODs and drugs for human use to determine whether the authorised products still benefited from an orphan status or not.

### Paediatric investigation plans

Of all authorised ODs, submitted PIPs including waivers, deferral agreements as well as description and timelines of the required studies (clinical, non-clinical and formulations) were extracted from the EMA website. As a rule, a PIP has to include all subsets of the paediatric population, but waivers for the entire paediatric population (full waivers) or for certain age groups (partial waivers) are granted when one of the following conditions are met: the condition only occurs in the adult population; clinical studies cannot be expected to be of significant therapeutic benefit or are not feasible; the product is considered unsafe or ineffective in children. Full waivers can be granted for classes of medicinal products (‘class waivers’) or for specific medicinal products (‘product specific waiver’).

In case of a deferral, the initiation or completion of paediatric studies described in the PIP is postponed until after MA for adults [1].

### Time course to marketing authorisation

The influence of the Paediatric Drug Regulation on the time course of obtaining MA after ODD was analysed using SPSS software version 22.0 (IBM Corp, Armonk, NY, USA). Chi-square was used for subgroup analyses and Cox proportional hazards models were used to examine the impact of potential paediatric use (intended for children yes/no) and approval after or before 2007 (the year in which the Paediatric Drug Regulation was introduced) on time to MA for a designated orphan indication. In order to analyse these effects, the database was restructured from a list of drugs with children and adult indications as separate variables to a table with drug- indication- age combinations. A drug with multiple indications for both children and adults can thus appear more than once in this table. ODD after or before 2007 was entered as separate variable. Time to event was computed as the time elapsed between ODD and MA for every drug-indication-age combination. Since a correlation can be assumed between obtaining authorisation for a paediatric indication and an adult indication for the same medicinal compound, the analysis was also repeated for drugs irrespective of indication by age. When designated orphan products were not authorised yet, the case was censored at the date of analysis.

The effect of repurposing could only be analysed for products with MA using the definition as described by Norman (2013) [10]. This variable was not available for drugs without MA (censored cases). Therefore this variable was not analysed with survival analysis but with General Linear Model (GLM) only. The mean time from ODD to MA was also calculated using the GLM procedure with the following covariates: after/before 2007 and paediatric indication (yes/no).

Differences were taken as significant at *P* < 0.05.

## Results

### Orphan drug designations and marketing authorisations

**Figure 1:**
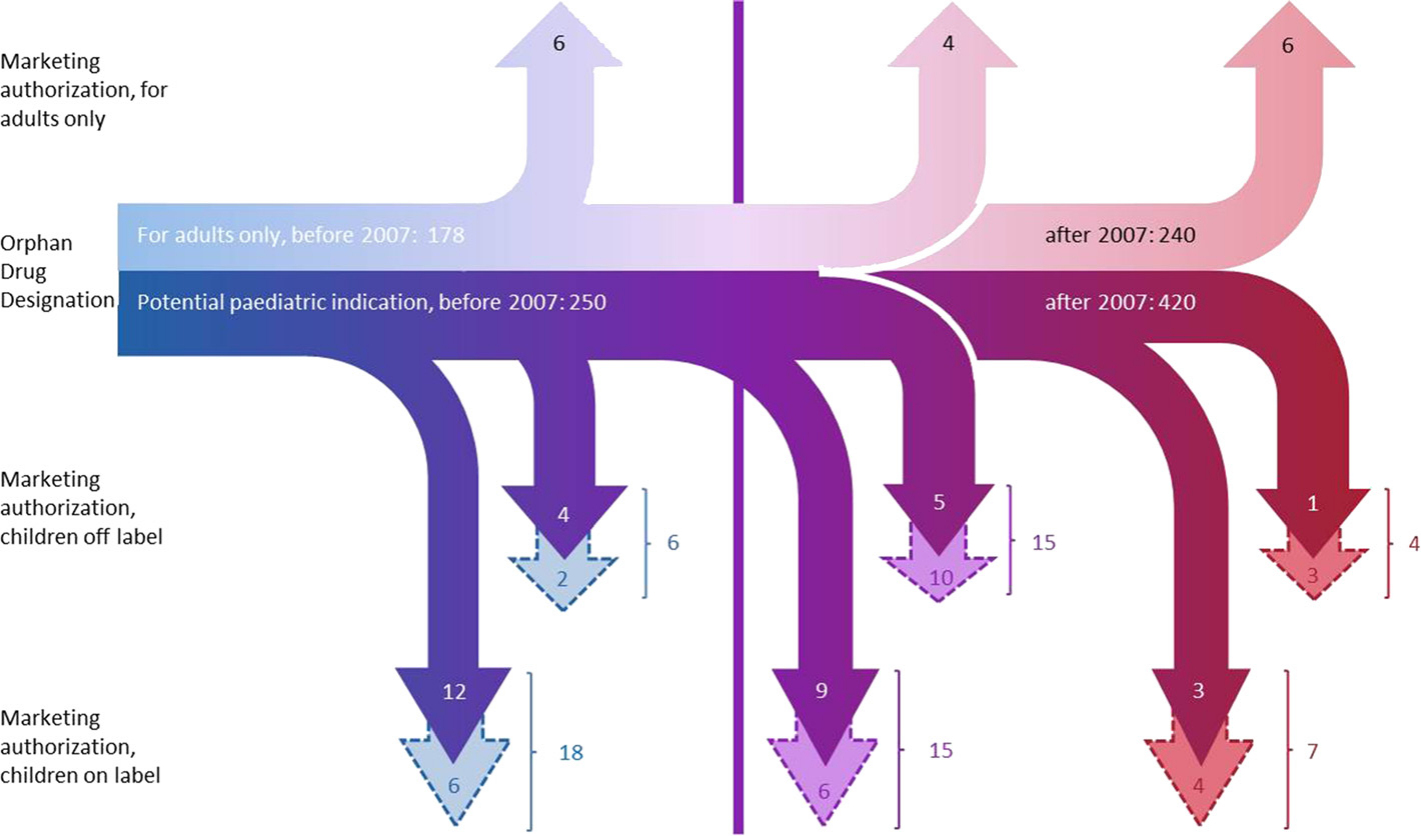
**Schematic overview of potential and authorised treatment populations.** The horizontal pipeline indicates the orphan drug designations (ODDs) for either adults only (upper line) or with a potential paediatric indication (lower line) over time (2000 – 2012) and the arrows represent those that obtained MA. The thick vertical line represents the year 2007. Arrows to the right of the thick line are all ODDs that obtained MA after 2007 (middle section: designation date before 2007, rightmost: designation date after 2007). Arrows with broken outline represent ODs that are undergoing further research in the paediatric population (i.e. with an agreed PIP after having received MA, while solid arrows are not undergoing further research in children).

From the implementation of the OD Regulation in 2000 until December 2012, 1088 ODDs were granted, 670 (62%) were intended for children (either exclusively for children (n = 161) or for both children and adults (n = 509)) and 418 (38%) were for adults only (Figure 1). As of November 2013, 81 of all granted ODDs had obtained MA. Sixty-five of these were identified as having a potential paediatric use at the time of ODD. Forty of these have indeed become available for children (‘on- label’); 25 potential paediatric products were still off label for children at the time of MA and 16 products were for adults only.

Of the 40 on-label paediatric ODs, 16 are currently under further development for a subset of the paediatric population. The PIP details of these ODs are specified in Table 2.

**Table 2:** PIP details of ODs that are authorised for use in children

**Medicine name (active substance)**	**Paediatric use**		**Paediatric investigation plan**			
	Potential paediatric*	**On label†**	**Decision**‡	**Condition and age covered by waiver**	**Ground for waiver**	**Expected date of completion §**
**Elaprase (idursulfase)**	Yes	All	PW	Mucopolysaccharidosis II (Hunter syndrome) (Girls birth to < 18 y)	Condition does not occur in the specified paediatric subset	December 2015
**Exjade¶ (deferasirox)**	Yes	2	PW	Chronic iron overload requiring chelation therapy (birth to < 2 years)	No significant therapeutic benefit	June 2015
Glivec* (imatinib)	Yes	1	PW	Pulmonary arterial hypertension (PAH) (birth to <6 months)	Likely ineffective	May 2013: PIP completed
				Philadelphia chromosome (BCR-ABL translocation) - positive chronic myeloid leukaemia (birth to <18 years)	No significant therapeutic benefit	
				Treatment of Philadelphia chromosome (BCR-ABL translocation) - positive acute lymphoblastic leukaemia (birth to < 1 year).	Condition does not occur in the specified paediatric subset	
**Ilaris (canakinumab)**	Yes	2	PW	Juvenile idiopathic arthritis (birth to < 24 months)	Condition does not occur in the specified paediatric subset and no significant therapeutic benefit	June 2015
				Cryopyrin Associated Periodic Syndromes (CAPS) including: FCAS, FCU, MWS, NOMID and CINCA* (birth to < 28 days)	No significant therapeutic benefit	
**Inovelon (rufinamide)**	Yes	4	PW	Lennox-Gastaut syndrome (birth to < 12 months and from 4 to <18 years)	Condition does not occur in the specified paediatric subset and no significant therapeutic benefit	September 2017
**Kuvan (sapropterin dihydrochloride)**	Yes	4	PW	Hyperphenylalaninemia (4 to < 18 years)	No significant therapeutic benefit	January 2014
**Mozobil (Plerixafor)**	Yes	All	PW	Myelosuppression caused by chemotherapy to treat malignant disorders, which requires an autologous haematopoietic stem cell transplant (birth to < 12 months )	No significant therapeutic benefit	June 2017
**Novothirtheen (catridecacog)**	Yes	6	PW	Prevention of bleeding during surgical interventions in congenital factor XIII A-subunit deficiency and treatment of bleeding in congenital factor XIII A-subunit deficiency (birth to <18 years)	Condition does not occur in the specified paediatric subset	December 2015
				For the prevention of bleeding in congenital factor XIII A-subunit deficiency (birth to <1 year)	No significant therapeutic benefit	
**Tobi Podhaler (tobramycin)**	Yes	6	PW	Pseudomonas aeruginosa pulmonary infection/colonisation in patients with cystic fibrosis (birth to < 3 months)	Likely unsafe and no significant therapeutic benefit	September 2015
**Tracleer¶ (bosentan monohydrate)**	Yes	3	PW	Systemic sclerosis and of interstitial pulmonary (birth to < 18 years)	Condition does not occur in the specified paediatric subset	December 2013
				Pulmonary arterial hypertension (PAH) (from 28 days to < 3 months and from 12 – 18 years)	No significant therapeutic benefit	
**Votubia (everolimus)**	Yes	3	PW	Angiomyolipoma (birth to < 18 years)	Condition does not occur in the specified paediatric subset	March 2020
				Subependymal giant cell astrocytoma and tuberous Sclerosis Complex (NA)	NA	
**Vpriv (velaglucerase alfa)**	Yes	3	PW	Gaucher Disease, Type 2 (birth to < 18 years)	Likely ineffective	July 2015
				Gaucher Disease, types 1 and 3 (from birth to < 24 months)	No significant therapeutic benefit	
**Xagrid¶ (anagrelide)**	Yes	All	PW	Essential Thrombocythaemiaa (birth to < 6 years)	Condition does not occur in the specified paediatric subset	March 2013
**Kalydeco (ivacaftor)**	Yes	6	FP	NA	NA	December 2016
**Orfadin¶**	Yes	All	FP	NA	NA	May 2013
**Revatio¶**	Yes	1	FP	NA	NA	July 2014

*Intended for the paediatric population at time of ODD (yes/no).

†Minimum age (in years) on SmPC at time of MA. All: age range not specified and/ or no age contraindication.

‡PIP decision granted by EMA: PW: partial waiver, FP: Full investigation plan, for the entire paediatric population. NA: Not applicable.

§Expected date of PIP completion for the remaining population.

¶Product was authorised before 2007 however the MAH applied for or had the intention to apply for an extension of the authorised indication. Consequently, pursuant to Article 8 of Regulation (EC) No 1901/2006, the MAH submitted a PIP.

FCAS: Familial Cold Autoinflammatory Syndrome; FCU: Familial Cold Urticaria; MWS: Muckle-Wells Syndrome; NOMID: Neonatal-Onset Multisystem Inflammatory Disease; CINCA: Chronic Infantile Neurological, Cutaneous, Articular Syndrome.

Of the 25 ODs that are authorised for adults but still off label for children, fifteen products are currently undergoing further development for use in children (Table 3), while the remaining ten are not. Two of those were granted a product specific waiver because the medicinal product did not represent a significant therapeutic benefit over existing treatment for paediatric patients while the remaining eight products did not have a PIP (see below for further details).

**Table 3:** PIP details of potential paediatric ODs that are off-label to children

**Medicine name (active substance)**	**Paediatric use**		**Paediatric investigation plan**			
	Pot paed*	**On label†**	**Decision‡**	**Condition and age covered by waiver**	**Ground for waiver**	**Expected date of completion**
**Signifor (pasireotide diaspartate)**	Yes	No	PSW	Pituitary dependent Cushing, overproduction of pituitary ACTH, pituitary dependant hyperadrenocorticism and the treatment of acromegaly and pituitary gigantism (birth to <18 y)	No significant therapeutic benefit	Not applicable
**Vyndaqel (Tafamidis)**	Yes	No	PSW	Neuropathic heredofamilial (birth to <18 y)	No significant therapeutic benefit	Not applicable
**Adcetris (brentuximab vedotin)**	Yes	No	PW	Hodgkin (birth to < 5 y); Anaplastic large cell lymphoma (birth to < 2y)	Both conditions do not occur in the specified paediatric subset	December 2018
**Bronchitol (mannitol)**	Yes	No	PW	Cystic Fibrosis with pulmonary disease (birth to <6y)	No significant therapeutic benefit	April 2011
**Cayston (aztreonam lysine)**	Yes	No	PW	Treatment of gram-negative endobronchial infection in bronchiectasis patients (birth to <18 y)	No significant therapeutic benefit	October 2016
				Treatment of Pseudomonas aeruginosa (PA) pulmonary infection/colonisation in patients with cystic fibrosis (CF) (birth to <3 months)	No significant therapeutic benefit	
**Dacogen (decitabine)**	Yes	No	PW	Acute myeloid leukaemia (birth to < 28 days)	No significant therapeutic benefit	July 2021
**Firazyr (icatibant)**	Yes	No	PW	ACE inhibitor-induced angioedema (birth to < 18)	No significant therapeutic benefit	December 2017
				Hereditary angioedema (birth to < 2 years	No significant therapeutic benefit	
**Glybera (alipogene tiparvovec)**	Yes	No	PW	Hyperchylomicronaemia (birth to < 2 years)	Likely unsafe	December 2021
**Iclusig (ponatinib)**	Yes	No	PW	Chronic myeloid leukaemia (birth to <1y)	Condition does not occur in the specified paediatric subset	December 2020
				Acute lymphoblastic leukaemia (birth to <1 y)	No significant therapeutic benefit	
**Nplate (romiplostim)**	Yes	No	PW	Disease-related thrombocytopenia in myelodysplastic syndrome (birth to <18 years)	Likely unsafe	December 2014
				Immune thrombocytopenia (birth to <1 y)	No significant therapeutic benefit	
**Plenadren (hydrocortisone)**	Yes	No	PW	Adrenocortical Insufficiency (6 years to < 18 y)	No significant therapeutic benefit	October 2016
**Revestive (Teduglutide)**	Yes	No	PW	Short bowel syndrome (birth to < 4 months)	No significant therapeutic benefit	February 2017
**Revolade (eltrombopag olamine)**	Yes	No	PW	Idiopathic Thrombocytopenia Purpura (birth to <1 y) Secondary thrombocytopenia: NA	Condition does not occur in the specified paediatric subset	December 2019
**Soliris (eculizumab)**	Yes	No	PW	Paroxysmal Nocturnal Haemoglobinuria (PNH) (birth to < 2 y)	Condition does not occur in the specified paediatric subset	June 2019
				STEC-HUS patients: NA AHUS: NA	Conditions do not occur in the specified paediatric subset	
**Sprycel§ (Dasatinib)**	Yes	No	PW	Philadelphia chromosome (BCR-ABL translocation)-positive chronic myeloid leukaemia (0-1y) and Philadelphia chromosome (BCR-ABL translocation)-positive acute lymphoblastic leukaemia (0-1y)	Condition does not occur in the specified paediatric subset	June 2018
**Sutent§**	Yes	No	PW	Gastro-intestinal stromal tumour (0-6y)	Condition does not occur in the specified paediatric subset	June 2014
**NexoBrid (concentrate of proteolytic enzymes enriched in bromelain)**	Yes	No	FP	NA	NA	March 2019

*Intended for the paediatric population at time of ODD.

†Paediatric use specified on SmPC at time of MA (yes/no).

‡PIP decision granted by EMA: CW: Class Waiver, PSW: product specific waiver W: full waiver in all subsets of the paediatric population, PW: partial waiver, FP: Full investigation plan, for the entire paediatric population. NA: Not applicable.

§Product was authorised before 2007, however the MAH applied or had the intention to apply for an extension of the authorised indication. Consequently, pursuant to Article 8 of Regulation (EC) No 1901/2006, the MAH submitted a PIP. FCAS: Familial Cold Autoinflammatory Syndrome; FCU: Familial Cold Urticaria; MWS: Muckle-Wells Syndrome; NOMID: Neonatal-Onset Multisystem Inflammatory Disease; CINCA: Chronic Infantile Neurological, Cutaneous, Articular Syndrome. STEC-HUS: Shiga-Toxin Producing Escherichia Coli Haemolytic Uremic Syndrome; AHUS: Atypical Haemolytic Uraemic Syndrome.

For the sixteen ODs for adults, four class waivers were granted for the following conditions: multiple myeloma (n = 2), myelofibrosis (n = 1) and chronic lymphocytic leukaemia (n = 1). Five product specific waivers were granted because the condition only occurs in the adult population (n = 3), because the medicinal product did not represent a significant therapeutic benefit over existing treatment for paediatric patients (n = 1) or because the product was likely to be unsafe in the paediatric population (n = 1). For four products, no PIP was found. One of these was authorised after 2007 but before the implementation of article 8 (in January 2009). Noteworthy is the fact that the potential indication of three products was initially considered for ‘adults only’ at time of ODD, but these products are currently undergoing paediatric investigations, meaning that they are considered to be of potential paediatric use after all (Table 4).

**Table 4:** Waiver conditions of ‘adults only’ OD

**Medicine name (active substance)**	**Paediatric use**		**Paediatric investigation plan**			
	Potential paediatric^*^	**On label**^**†**^	**Decision‡**	**Condition and age covered by waiver**	**Ground for waiver**	**Expected date of completion PIP for remaining population**
**Arzerra (ofatumumab)**	No	No	CW	Chronic lymphocytic leukaemia (birth to <18y)	Class waiver	NA
**Imnovid (pomalidomide)**	No	No	CW	Multiple myeloma (birth to <18y)	Class waiver	NA
**Jakavi (ruxolitinib)**	No	No	CW	Myeolofibrosis (birth to <18y)	Class waiver	NA
**Thalidomide Celgene (thalidomide)**	No	No	CW	Multiple myeloma (birth to <18y )	Class waiver	NA
**Afinitor (everolimus)**	No	No	PSW	Renal cell carcinoma and pancreatic neuroendocrine tumour (birth to <18y)	Condition occurs only in adult populations	NA
**Esbriet (pirfenidone)**	No	No	PSW	Idiopathic Pulmonary Fibrosis (birth to <18y)	Condition occurs only in adult populations	NA
**Nexavar**^§^**(Sorafenib)**	No	No	PSW	Differentiated thyroid cancer (birth to <18y)	No significant therapeutic benefit over existing treatments for paediatric patients.	NA
**Revlimid (lenalidomide)**	No	No	PSW	Multiple myeloma and myelodysplastic syndromes (birth to <18 y)	Likely unsafe	NA
**Torisel (temsirolimus)**	No	No	PSW	For the treatment of mantle-cell lymphoma for all subsets of the paediatric	Condition occurs only in adult populations	NA
**Bosulif (bosutinib)**	No	No	PW	Chronic myeloid leukaemia (birth to <10 y)	Condition occurs only in adult populations	December 2016
**Tasigna (Nilotinib)**	No	No	PW	Gastro-intestinal stromal tumour (0-18y) and chronic myeloid leukaemia (0-1y)	No significant therapeutic benefit	September 2015
**Volibris (ambrisentan)**	No	No	PW	Pulmonary arterial hypertension (0-1y)	Likely unsafe	December 2016

^*^Intended for the paediatric population at time of ODD (yes/no).

†Paediatric use specified on SmPC at time of MA.

^‡^PIP decision granted by EMA: CW: Class Waiver , PSW: product specific waiver; PW: partial waiver; NA: Not applicable.

^§^Product was authorised before 2007, however the MAH applied or had the intention to apply for an extension of the authorised indication. Consequently, pursuant to Article 8 of Regulation (EC) No 1901/2006, the MAH submitted a PIP.

### Paediatric investigation plans

For 36 authorised ODs no decision or information about a PIP was found. For the majority of the products a PIP was not required because approval was granted before the Paediatric Drug Regulation came into force (n = 19) or because application for MA was submitted before the implementation of article 7 (n = 4) or article 8 (n = 1). Unless the applicant files for extension or variation of the initial MA, these medicinal products are likely to remain off label to children. The remaining 12 products without a PIP were developed for (a subgroup of) children.

For 34 authorised ODs, the PIP was required to include development and testing of an age appropriate formulation or conducting non-clinical and clinical studies. Most of these (30/34) were granted a partial waiver, the remaining four products were required to develop and assess treatment for the complete paediatric population. None of the PIPs were completed at the time of application for MA as some of the requirements in the PIP were deferred. Partial waivers were mostly granted based on the expectation that clinical studies would be of no significant therapeutic benefit or fulfil no therapeutic need of the paediatric population. PIP decisions, waiver conditions and expected date of PIP completion are described in Tables 2, 3 and 4.

Half of the 34 products with a PIP were required to either develop an age-appropriate formulation or to assess the acceptability of the existing formulation (Table 5). The majority of these measures applied to oral formulations (n = 13). An age-appropriate diluted formulation was required for intravenous (n = 1) and subcutaneous (n = 2) formulations.

**Table 5:** Studies agreed upon in the PIPs of ODs

**Measure**	**N**
Quality	
- Development of age appropriate formulation	14
- Assessment of acceptability/ palatability	2
- Bioequivalence	1
- Microbiological testing	2
**Total**	**19**
Non-clinical	
- Juvenile toxicity study	20
- Other	8
**Total**	**28**
Clinical	
- Meta-analysis	1
- Randomised, double blind, placebo controlled	25
- Comparative, open label	20
- Uncontrolled	41
- Observational	3
- Bioequivalence/ bioavailability	5
- (PB)PK	2
- Pooled data	3
- Extrapolation	3
- Other	1
**Total**	**104**

For 15 products non-clinical studies had to be performed. The required measures mostly included juvenile animal studies to determine pharmacokinetics, tolerability, toxicology and/ or toxicokinetics. In some cases, specific pharmacology, exploratory or dose ranging studies were required in vitro or in other animal models.

All 34 products with a PIP required at least one clinical study in children (median = 3, range 1–9 studies). A quarter (n = 25) of the studies were randomised double-blind, placebo controlled studies in the target population. Another 20 studies were open label comparative trials and were either dose-comparative or using an active comparator, historical controls or standard care as controls. The majority of studies were, however, uncontrolled or observational “all-in-one” trials gathering as much data as possible in the target paediatric population, including efficacy, safety, tolerability, activity and/or pharmacokinetics (Table 5).

To date, only one orphan medicinal product completed its PIP (Glivec®), all other PIPs are still on-going. For four products the therapeutic indication has been extended to the paediatric population [15],[16]. On average, it takes seven years before PIPs are expected to be completed.

### Time course to marketing authorisation

**Figure 2:**
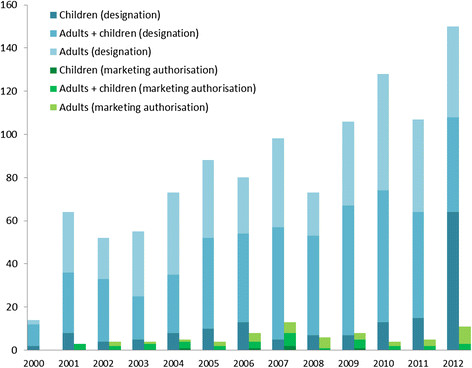
New orphan drug designation and marketing authorisations per year and age category.

Figure 2 illustrates the ODDs and MAs per year. The Paediatric Drug Regulation did not significantly increase the number of ODDs with potential paediatric indications (250/428 versus 420/660 of ODDs, χ^2^ = 2.78, p = 0.1) and did not lead to more MAs for ODs for children (18/30 vs 22/51 of MAs, χ^2^ = 1.53, p = 0.22). Table 6 summarises the indication, age range and authorisation details of MAs for use in the paediatric population.

**Table 6:** All ODs with MA for the paediatric population

**Medicine name**	**Active substance**	**Indication**	**Age range**	**Authorisation date**	**Status**
**Fabrazyme**	Agalsidase beta	Fabry disease (galactosidase-A deficiency)	8 years and older	03/08/2001	End of marketing exclusivity
**Replagal**	Agalsidase alpha	Fabry disease (galactosidase-A deficiency)	7 years and older	03/08/2001	Authorised under exceptional circumstance, end of marketing exclusivity
**Glivec**	Imatinib	Chronic myeloid leukaemia	> 1 and >2 years	07/11/2001	Withdrawn OD status
**Tracleer**	Bosentan monohydrate	Pulmonary arterial hypertension (PAH)	2 years and older	15/05/2002	Authorised
**Zavesca**	Miglustat	Niemann-Pick type-C disease	Children* and adults	20/11/2002	Authorised
**Carbaglu**	Carglumic acid	Hyperammonaemia due to - N-acetylglutamate-synthase (NAGS) primary deficiency	As early as the first day of life	24/01/2003	Authorised, end of marketing exclusivity for NAGS
		- isovaleric acidaemia			
		- methymalonic acidaemia			
		- propionic acidaemia			
**Aldurazyme**	Laronidase	Mucopolysaccharidosis I (alpha-L-iduronidase deficiency)	Children* and adults	10/06/2003	Authorised under exceptional circumstance, end of marketing exclusivity
**Busilvex**	Busulfan	Conditioning treatment prior to conventional haematopoietic progenitor cell transplantation (HPCT)	Newborn and older	09/07/2003	End of marketing exclusivity
**Lysodren**	Mitotane	Advanced adrenal cortical carcinoma	Children** and adults	28/04/2004	Authorised
**Pedea**	Ibuprofen	Patent ductus arteriosus	Premature newborns	29/07/2004	Authorised
**Wilzin**	Zinc	Wilson’s disease	One year and older	13/10/2004	Authorised
**Xagrid**	Anagrelide	Essential thrombocythaemia	Children** and adults	16/11/2004	Authorised under exceptional circumstances
**Orfadin**	Nitisinone	Hereditary tyrosinaemia type 1 (HT-1)	Children* and adults	21/02/2005	Authorised
**Revatio**	Sildenafil	Pulmonary arterial hypertension	one year and older	28/10/2005	Authorised
**Naglazyme**	Galsulfase	Mucopolysaccharidosis VI (N-acetylgalactosamine-4-sulfatase deficiency; Maroteaux-Lamy syndrome)	Children* and adults	24/01/2006	Authorised under exceptional circumstances
**Myozyme**	Alglucosidase alpha	Pompe disease (acid-α-glucosidase deficiency)	Children of all ages and adults	29/03/2006	Authorised
**Evoltra**	Clofarabine	Acute lymphoblastic leukaemia	1-21 years	29/05/2006	Authorised under exceptional circumstances
**Exjade**	Deferasirox	Beta thalassaemia major with iron overload	2 years and older	28/08/2006	Authorised
**Diacomit**	Stiripentol	Severe myoclonic epilepsy in infancy (SMEI, Dravet's syndrome)	3 years and older	04/01/2007	Conditional approval
**Elaprase**	Idursulfase	Hunter syndrome (mucopolysaccharidosis II)	5 years and older	08/01/2007	Authorised under exceptional circumstances
**Inovelon**	Rufinamide	Lennox-Gastaut syndrome	4 years and older	16/01/2007	Authorised
**Cystadane**	Betaine anhydrous	Homocystinuria	Children* and adults	15/02/2007	Authorised
**Soliris**	Eculizumab	Paroxysmal nocturnal haemoglobinuria (PNH) and atypical haemolytic uremic syndrome (aHUS)	Children* and adults	20/06/2007	Authorised
**Siklos**	Hydroxycarbamide	Sickle-cell syndrome	2 years and older	29/06/2007	Authorised
**Increlex**	Mecasermin	Primary insulin-like-growth-factor-1 deficiency (primary IGFD)	2 to 18 years	03/08/2007	Authorised under exceptional circumstances
**Atriance**	Nelarabine	Acute lymphoblastic leukaemia (T-ALL) and T-cell lymphoblastic lymphoma (T-LBL)	Children* and adults	22/08/2007	Authorised under exceptional circumstances
**Kuvan**	Sapropterin dihydrochloride	Phenylketonuria (PKU) and tetrahydrobiopterin (BH4) deficiency	4 years and older	02/12/2008	Authorised
**Mepact**	Mifamurtide	Osteosarcoma	2 to 30 years	06/03/2009	Authorised
**Peyona**	Caffeine citrate	Primary apnoea	Premature newborns	02/07/2009	Authorised
**Mozobil**	Plerixafor	Lymphoma and multiple myeloma	Children** and adults	31/07/2009	Authorised
**Cayston**	Aztreonam lysine	Cystic fibrosis (CF)	6 years and older	21/09/2009	Authorised
**Ilaris**	Canakinumab	Cryopyrin-Associated Periodic Syndromes (CAPS), and Systemic Juvenile Idiopathic Arthritis (SJIA)	2 years and older	23/10/2009	Authorised under exceptional circumstances, withdrawn OD status
**Tepadina**	Thiotepa	Allogeneic or autologous haematopoietic progenitor cell transplantation (HPCT)	Children* and adults	15/03/2010	Authorised
**Vpriv**	Velaglucerase alpha	Type-1 Gaucher disease	>2 years	26/08/2010	Authorised
**Tobi Podhaler**	Tobramycin	Cystic fibrosis	6 years and older	20/07/2011	Authorised
**Votubia**	Everolimus	Subependymal giant-cell astrocytoma (SEGA) associated with tuberous-sclerosis complex (TSC)	3 years and older	02/09/2011	Conditional approval
**Xaluprine**	6-Mercaptopurine monohydrate	Acute lymphoblastic leukaemia (ALL)	Children* and adults	09/03/2012	Authorised
**Kalydeco**	Ivacaftor	Cystic fibrosis (CF) with G551D mutation in the CFTR gene	6 years and older	23/07/2012	Authorised
**Novothirteen**	Catridecacog	Congenital factor-XIII-A-subunit deficiency	6 years and above	03/09/2012	Withdrawn OD status
**Procysbi**	Mercaptamine bitartrate	Nephropathic cystinosis	Children* and adults	06/09/2013	Authorised
**Orphacol**	Cholic acid	Inborn errors in primary bile-acid synthesis due to 3-hydroxy-5-C27-steroid oxidoreductase deficiency or 4-3-oxosteroid-5-reductase deficiency	One month and older	12/09/2013	Authorised under exceptional circumstances
**Defitelio**	Defibrotide	Severe hepatic veno-occlusive disease (VOD) in haematopoietic stem-cell transplantation (HSCT) therapy	One month and older	18/10/2013	Authorised under exceptional circumstances

*Age range not specified in SmPC.

**Not contraindicated in children, however the SmPC mentions a special warning (such as “The effects of medicinal product on children and adolescents have not been studied” or “limited information on the use in children”).

**Figure 3:**
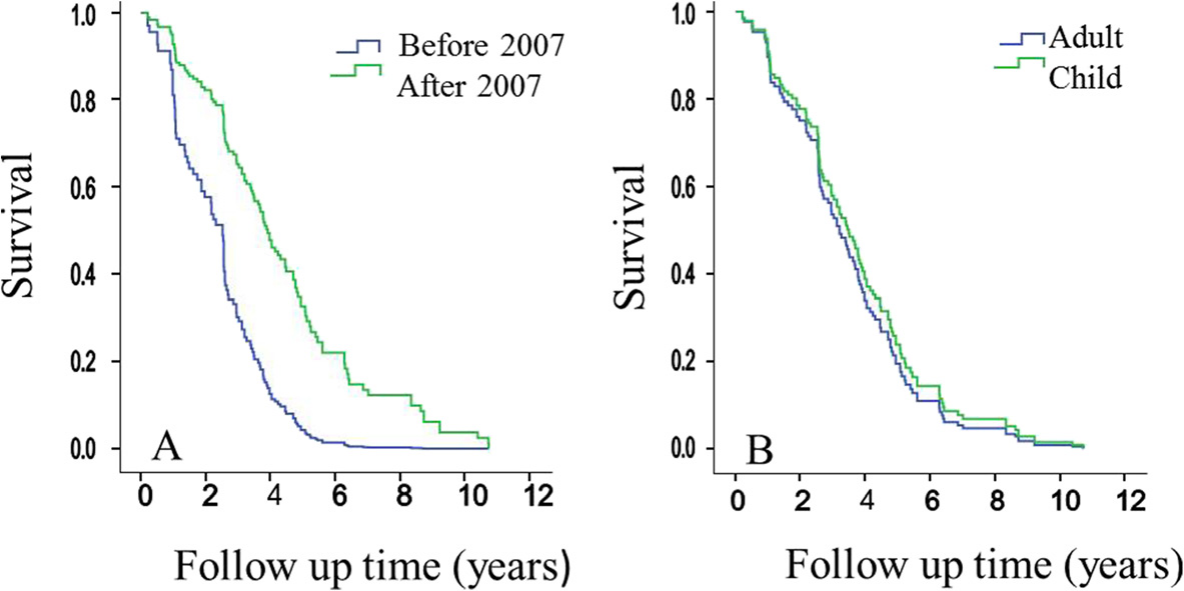
Cox regression survival curve as a function of (A) 2007 (after/ before) and (B) age group (child/ adult).

The final model to analyse the time between ODD and MA as survival time included after/before 2007 and age group (child/ adult) as categorical variables. The results show that after the implementation of the Paediatric Drug Regulation in 2007, drug-indication-age combinations, have a longer time to authorisation than before January 2007 (Hazard ratio (95% CI) 2.804 (1.837-4.280), p < 0.001, Figure 3A). The same effect was observed when multiple indications of one drug were grouped (data not shown). Potential paediatric use did not prolong the overall drug development process compared to ‘adults only’ medicinal products (Hazard ratio (95% CI) 1.140 (0.767-1.696), p = 0.52, Figure 3B).

The mean (95% CI) time to authorisation for paediatric medicinal products after and before 2007 was 4.04 (3.02-5.07) and 2.93 (1.93-3.92). The mean (95% CI) time to authorisation for adults-only products after and before 2007 was 4.45 (3.78-5.12) and 2.07 (1.52-2.63) years.

Repurposing does not provide any benefit in shortening the authorisation process for neither paediatric nor adult ODs (p = 0.21, GLM, data not shown).

## Discussion

More than 80 ODs, covering nearly 100 indications, were authorised in Europe since 2000. Half of these products are available for (a subgroup of) children. Another 34 authorised ODs are currently undergoing further investigations in children. The introduction of the Paediatric Drug Regulation was associated with a longer time to MA for OD, did not significantly increase the number of ODDs with potential paediatric indications and did not lead to more MAs for paediatric indications.

In this study we were able to quantify the time to authorisation and the number of paediatric ODs, but could not extract the quality of research conducted in children given the relatively young EU Paediatric Drug Regulation. The use of Cox regression to analyse time to MA as a survival function is appropriate and the data set is large enough to draw valid conclusions. There is some autocorrelation between indications for children and adults within the same drug. This means that the time to MA for a paediatric indication is linked to that for adult indications of the same drug, because, in part, they share study results. The data set illustrates that ODs often obtain MA for adults first, for which clinical studies are easier to conduct, and later for children. A control group would have been desirable, but since non-ODs do not have the same starting point (time of obtaining ODD), comparison in this context is not possible and data would have to be based on different criteria which is beyond the scope of this study.

Administrative processes are not static, they change over time, and that also applies to the approval of ODs. This implies that the time to MA, modelled as survival time in our Cox regression model, may not be completely independent of time. This time dependency was addressed by using after/before 2007 as a separate categorical variable. Since the granted therapeutic indication at the time of MA is the result of the assessment of the quality, safety and efficacy data submitted with the marketing application, this may be different (narrower) to the indications proposed at the time of ODD application [17]. After 2007, the Paediatric Committee (PDCO) safeguards that for any potential paediatric medicinal product an investigation plan is made.

The situation for patients with rare diseases has, without a doubt, improved dramatically after 2000, the year in which the EU Orphan Drug Regulation was implemented. Before 2000 only eight products, so called orphan-like drugs, were authorised for the treatment of rare diseases with the support of the EMA [18]. Four of these orphan-like drugs were authorised for use in children. In contrast, the United States introduced the Orphan Drug Act almost 20 years earlier, in 1983 [19]. Over the period 2000–2009, 148 (13%) of 1138 ODDs received MA in the U.S., of which 81% were potentially beneficial for children [20]. In the same period in the EU only 55 of 703 of ODDs were authorised and only 52% of the products were authorised in children.

The Paediatric Drug Regulation, implemented to increase the availability of effective and safe drugs of good quality for children, was also beneficial for ODs. The majority of ODs with potential paediatric use that were off label to children at the time of MA (40% of all ODs) is currently in development for the paediatric population. Also, 40% of ODs authorised for children are undergoing further investigations to either expand the intended treatment group to include younger children and/or to develop an age appropriate formulation for youngsters. This would presumably not have taken place without the instalment of the Paediatric Drug Regulation.

A drawback is the high number of deferrals for both ordinary and orphan products. In deferrals, either initiation or completion of paediatric studies is postponed until the medicinal product is authorised for use in adults, to ensure that it is safe to do research in children and that availability for adults is not delayed.

In the 5 year progress report on the paediatric regulation, the EMA concluded that authorisation of medicines for adults was not delayed. However, in our analysis, products authorised before 2007 had a shorter time to MA than those authorised after the Paediatric Drug Regulation came into force. Apparently the Paediatric Drug Regulation added complexity to the R&D and regulatory process of orphan medicinal products, exemplified by the applicants’ investments time and effort in drafting a PIP.

Others also expressed concern that the EU Paediatric Drug Regulation retards drug development and authorisation for adults by demanding paediatric trials, especially for rare diseases [21],[22]. It is not only the Paediatric Drug Regulation that causes delay. There are potential other product- and company-related factors such as the indication for which a drug is being developed, the type of drug product in development, the company’s experience in developing OD and the size of the companies submitting the MA application [23]–[25], and incentives such as those for Small and Medium Sized companies implemented in December 2005 [26]. Other economic and bureaucratic issues such as the increasing amount of regulations where applicants have to comply with during drug development in general have their effects. However, the increased approval time after 2007 can also be an artefact, caused by the submission of ODD applications increasingly earlier in the developmental phase.

Given the relatively young EU Paediatric Drug Regulation, there are few data on PIP completion and outcome in rare diseases, especially since deferrals lead to an additional seven years before expected completion of the files for paediatric indications. So far, only one product successfully reached completion of development. Furthermore, no orphan-designated medicine has yet obtained the orphan incentive of two additional years of market exclusivity. The impact of introducing PIPs will become apparent in the next few years when more PIPs are expected to be completed and will learn whether applicants are compliant with measures and timelines agreed upon in PIPs.

We could not demonstrate that repurposing is an effective strategy for the development of drugs for rare diseases in children. Drug repurposing is considered an interesting acceleration and facilitation of OD development at lower cost and with lower risk of failure, since these drugs have already been studied [7]. Although repurposed drugs have already been studied in animals and/or humans to some extent, a positive benefit/risk balance has to be established for the intended paediatric population. Since research in children on average takes another seven years after safety and efficacy have been confirmed in adults, this is considered to be the rate-limiting step, irrespective of repurposing.

There is a need for novel research tools to support decisions that balance between exposing children to experiments and the obvious need to provide children with authorised good quality drugs. Comparative trials are considered the primary instrument to collect the evidence needed for MA. However, for rare disease this is often not feasible. In most cases, the studies requested in the PIPs were open label uncontrolled studies. Most studies were designed to collect as much data as possible, ranging from pharmacokinetics and dose finding to safety and efficacy. When experimental research is not feasible, on-going data collection through registry/observational programs (such as named patient programmes (NPP) or compassionate use) are in place to characterise both long-term safety and efficacy as well as to determine patient characteristics and disease progression [27],[28]. There are differences in implementation of legislation throughout the EU. For example, the French authorities explicitly mention that investigation is not the goal of an NPP and that an NPP may not replace a clinical trial [29].

Several novel research strategies have been proposed, such as meta-analytic approaches, extrapolation, modelling and simulation. With the use of sparse sampling, population pharmacokinetics/pharmacodynamics (POP-PK/PD) and/or physiologically based pharmacokinetic models, extrapolation from adults to children, interpolation between paediatric age subgroups and the optimal use of scientific literature and in vitro/preclinical data, drug development is enriched while minimising the burden of studies in children [30]. Since the implementation of the Paediatric Drug Regulation, especially simulation and modelling are increasingly used for paediatric drug development [15].

Non-clinical juvenile studies are often used to bridge the knowledge gap between mature and immature systems, to detect safety issues early and to predict the dose in children. A recent survey showed that in the majority of juvenile toxicity studies, findings were comparable to those for adults, yielding no new information [31]. Furthermore, novel toxicity was uncommon and could have been predicted from either known pharmacology or from adult data. On the other hand, in a preliminary review of 5 completed juvenile animal studies required in PIPs, unexpected organ toxicity and increased sensitivity was observed in 3 medicinal products, stressing the importance of conduction juvenile animal studies [15]. It confirms that drug development is not a “one size fits all” process. A case-by-case evaluation process is necessary, especially for paediatric ODs.

Part of paediatric drug development is to avoid duplication and to ensure that ongoing and planned paediatric research is transparent. To this purpose, in March 2011, the EU Clinical Trials Register (EU-CTR) was made publicly accessible (EU-CTR) for paediatric trials included in a PIP [15]. The website (available at https://www.clinicaltrialsregister.eu) provides public access to information extracted from the EU clinical trials database (EudraCT), such as protocols and known results. The clinical trials included are those with agreed PIPs from investigator sites within and outside the European Economic Area (EEA). As soon as a paediatric trial is approved, it becomes accessible in the database.

## Conclusions

The EU Paediatric Drug Regulation did not increase the number of ODDs with potential paediatric indications nor did it lead to more MAs for paediatric indications. It was associated with a longer time to MA for both adult and paediatric orphan indications. Nonetheless, the Paediatric Drug Regulation has ensured the further paediatric development of drugs still off-label to children. The impact on the quality and volume of research in the paediatric population through PIPs will become clear in the coming few years. Case-by-case assessment, based on innovative research tools is necessary to collate the best evidence while protecting children from unnecessary experiments.

## Abbreviations

CW: Class waiver

EEA: European economic area

EMA: European medicines agency

EPAR: European public assessment report

EU: European union

EU-CTR: EU clinical trials register

EudraCT: EU clinical trials database

FDA: US food and drug administration

FP: Full PIP for the entire paediatric population

GLM: General linear model

MA: Marketing authorisation

NA: Not applicable

NPP: Named patient programme

OD: Orphan drug

ODD: Orphan drug designation

PDCO: Paediatric committee

PIP: Paediatric investigational plan

POP-PK/PD: Population pharmacokinetics/pharmacodynamics

PSW: Product specific waiver

PUMA: Paediatric use marketing authorisation

PW: Partial waiver

SmPC: Summary of product characteristics

W: Full waiver in all subsets of the paediatric population

## Competing interests

ACE Pharmaceuticals BV specialises in medical need products and orphan drugs. The authors declare that they have no competing interests.

## Authors’ contributions

ARKV and PJdV designed the study, collected data and performed analysis and interpretation of the data. RvdV and AdB were involved in writing and revising the manuscript. All the authors read and approved the final manuscript.
